# Implementation fidelity of a strategy to integrate service delivery: learnings from a transitional care program for individuals with complex needs in Singapore

**DOI:** 10.1186/s12913-019-3980-x

**Published:** 2019-03-19

**Authors:** Milawaty Nurjono, Pami Shrestha, Ian Yi Han Ang, Farah Shiraz, Joanne Su-Yin Yoong, Sue-Anne Ee Shiow Toh, Hubertus Johannes Maria Vrijhoef

**Affiliations:** 1 Centre for Health Services Research and Policy Research, Saw Swee Hock School of Public Health, National University of Singapore, National University Health System, Singapore, Singapore; 2 Regional Health System Planning Office, National University Health System, Singapore, Singapore; 3 Saw Swee Hock School of Public Health, National University of Singapore, Singapore, Singapore; 4 Center for Economic and Social Research, University of Southern California, Los Angeles, USA; 5 Yong Loo Lin School of Medicine, National University of Singapore, National University Health System, Singapore, Singapore; 6 Department of Patient and Care, University Hospital Maastricht, Maastricht, The Netherlands; 7 Department of Family Medicine and Chronic Care, Vrije Universiteit Brussels, Brussels, Belgium; 8 Panaxea B.V, Amsterdam, The Netherlands

**Keywords:** Integrated care, Post-discharge care, Transitional care, Implementation fidelity, Mixed methods

## Abstract

**Background:**

To cope with rising demand for healthcare services in Singapore, Regional Health Systems (RHS) comprising of health and social care providers across care settings were set up to integrate service delivery. Tasked with providing care for the western region, in 2012, the National University Health System (NUHS) – RHS developed a transitional care program for elderly patients with complex healthcare needs who consumed high levels of hospital resources. Through needs assessment, development of personalized care plans and care coordination, the program aimed to: (i) improve quality of care, (ii) reduce hospital utilization, and (iii) reduce healthcare-related costs. In this study, recognizing the need for process evaluation in conjunction with outcome evaluation, we aim to evaluate the implementation fidelity of the NUHS-RHS transitional care program to explain the outcomes of the program and to inform further development of (similar) programs.

**Methods:**

Guided by the modified version of the Conceptual Framework for Implementation Fidelity (CFIF), adherence and moderating factors influencing implementation were assessed using non-participatory observations, reviews of medical records and program databases.

**Results:**

Most (10 out of 14) components of the program were found to be implemented with low or moderate level of fidelity. The frequency or duration of the program components were observed to vary based on the needs of users, availability of care coordinators (CC) and their confidence. Variation in fidelity was influenced predominantly by: (1) complexity of the program, (2) extent of facilitation through guiding protocols, (3) facilitation of program implementation through CCs’ level of training and confidence, (4) evolving healthcare participant responsiveness, and (5) the context of suboptimal capability among community providers.

**Conclusion:**

This is the first study to assess the context-specific implementation process of a transitional care program in the context of Southeast Asia. It provides important insights to facilitate further development and scaling up of transitional care programs within the NUHS-RHS and beyond. Our findings highlight the need for greater focus on engaging both healthcare providers and users, training CCs to equip them with the relevant skills required for their jobs, and building the capability of the community providers to implement such programs.

**Electronic supplementary material:**

The online version of this article (10.1186/s12913-019-3980-x) contains supplementary material, which is available to authorized users.

## Background

Around the world, healthcare systems are experiencing significant pressures in coping with rapidly aging populations and growing multi-morbidity. Like other developed countries, Singapore’s population is also aging quickly, accompanied by the mounting prevalence of co-morbidities [1]. Multi-morbidities increase complexity in service delivery, requiring high-level support from different professionals spanning different care settings [2]. Historically, healthcare services in Singapore have been provided largely in silos, with little coordination across different parts of the system [3]. Such fragmentation resulted in over-utilization, including excess hospitalization and service duplication, leading to suboptimal system efficiency, medical errors, increased costs, and low patient satisfaction [4, 5]. Even after the addition of new hospitals, Singapore’s healthcare system continues to struggle with high bed occupancy rates and long waiting times at acute hospitals [6], indicating a need to improve integration across care settings.

Since 2011, the Regional Health Systems (RHSs) have been established in various geographical regions in Singapore to build partnerships between different care providers, within and across care settings. Led by the acute hospital within each region, each RHS aims to foster integration and deliver seamless care for the population within the respective catchment areas in a cost-effective way [7, 8].

Among all patients cared for under the RHS, frequent admitters (FAs) (patients who have had three or more hospital admissions in a year) consume a disproportionally high share of healthcare resources. In 2013, within the National University Health System (NUHS)-RHS, FAs accounted for only 1% of all patients, yet generated about 27% of all inpatient episodes and incurring higher average costs compared to those who were not FAs [9]. A sub-optimal transition from hospital to home and community was identified as a key contributing factor of hospital re-admissions [10]. In response to such needs, the NUHS-RHS prioritized efforts to improve the transition following discharge from hospital and anchor care in the community as part of integrated service delivery.

Transitional care is a set of multi-disciplinary interventions aimed at improving care coordination when patients transit between different healthcare settings [11]. Discharge planning [12], case management [13], home visits [14], telephone follow-ups [15], a combination of both home visits and telephone monitoring [14], and hospital-to-home transitional care as a whole [16–18] have all been shown to be effective in reducing healthcare utilization.

**Fig. 1:**
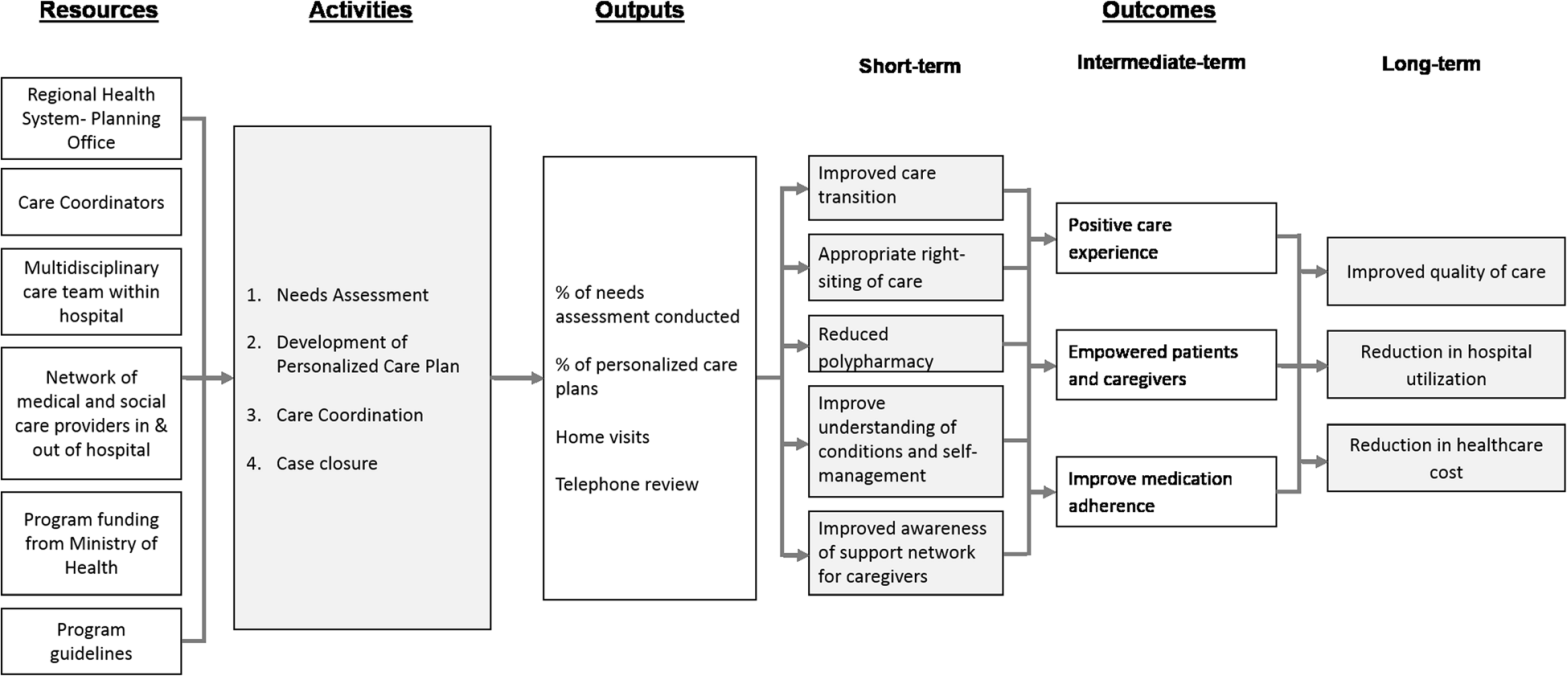
The logic model of NUHS-RHS transitional care program

In 2012, the NUHS-RHS initiated a transitional care program for elderly patients and/or those with complex healthcare needs who had previously consumed high levels of hospital resources [19]. Elderly patients and FAs with multiple chronic conditions, limited ambulation and caregiver(s) at home were eligible for the program. As depicted in Fig. 1, through comprehensive needs assessment, development of personalized care plans with multi-disciplinary inputs, and care coordination, the program aimed to: (i) improve quality of care, (ii) reduce hospital utilizations, and (iii) reduce healthcare related cost. Patients who fit the program selection criteria were enrolled into the program for a limited duration (3 months or 12 months) depending on the results of the needs assessment.

To facilitate further development of the NUHS transitional care program, a rigorous and practical evaluation strategy was required to examine progress and identify insights for improvement and scaling-up. One way to achieve this was by assessing implementation fidelity. Implementation fidelity, the degree in which a program is implemented as intended, acts as a potential mediator of the relationship between programs and their intended outcomes [20]. Higher implementation fidelity is associated with increased likelihood of success [21]. For this reason, evaluation of implementation fidelity has been increasingly promoted in conjunction with outcome evaluation to discern the true effects of healthcare programs by taking into consideration variation in implementation [21–23]. However, in the context of Singapore, most of the previously conducted evaluation on transitional care programs [18, 24–26] focused mainly on assessing the effectiveness with limited information on the direct implementation processes, making it difficult to make program improvements.

To address this gap, we aimed to evaluate the implementation fidelity of the NUHS-RHS transitional care program to explain the outcomes of the program. The program had been demonstrated to result in lower mortality among FAs and higher healthcare utilization and cost compared to their matched controls [27].

Furthermore, the study also aim to inform further development of the program. Since this gap is present in other countries too, this study may provide relevant insights to those involved in the development, implementation and/or evaluation of transitional care programs as part of integrated care initiatives beyond Singapore.

## Methods

### Conceptual framework

The modified version of the Conceptual Framework of Implementation Fidelity (CFIF) was used in this study [22]. The modified CFIF defines implementation fidelity as a measure of adherence to the intent of a program. Adherence takes into account the content and dose (frequency, duration, and coverage) of the program. The framework proposes that the level of fidelity is influenced by moderating factors including participant responsiveness, program complexity, comprehensiveness of policy description, strategies to facilitate implementation, quality of delivery, recruitment, and context, which are often inter-connected to each other.

Participant responsiveness refers to how well participants (users receiving the program and providers delivering program) respond to, or are engaged by the program. If participants do not value the program, they will not be engaged in the program, thereby making implementation difficult. Program complexity refers to the comprehensiveness of the program as evaluated through descriptions of the program and the actual implementation. Simple but specific programs were found to be more likely implemented with high fidelity than complex and/or vaguely described programs. Adequate facilitation is expected to optimize fidelity. Quality of delivery describes whether a program is delivered appropriately to achieve what was intended. A poorly delivered program contributes to lower implementation fidelity overall. In assessing participant recruitment, the modified CFIF proposes to assess the reasons for non-participation among potential participants and subgroups that were less likely to participate. Finally, context considers surrounding social system, for example social structures and cultures.

### Intervention: The NUHS transitional care program

Guided by a set of selection criteria, eligible patients were recruited mainly from the inpatient wards through referrals from the physicians, nurses and other allied health professionals. Then, needs assessments were conducted and personalized care plans were developed accordingly. One or a group of dedicated CCs was assigned to every patient enrolled into the program. Every CC assumed the role of an integrator, responsible for coordinating the delivery of services through communication with patients, their families, and the various professionals and services that the healthcare users come into contact with throughout their period of enrolment in the program. The transitional care program started after patients were discharged from the hospital.

### Study design

**Fig. 2:**
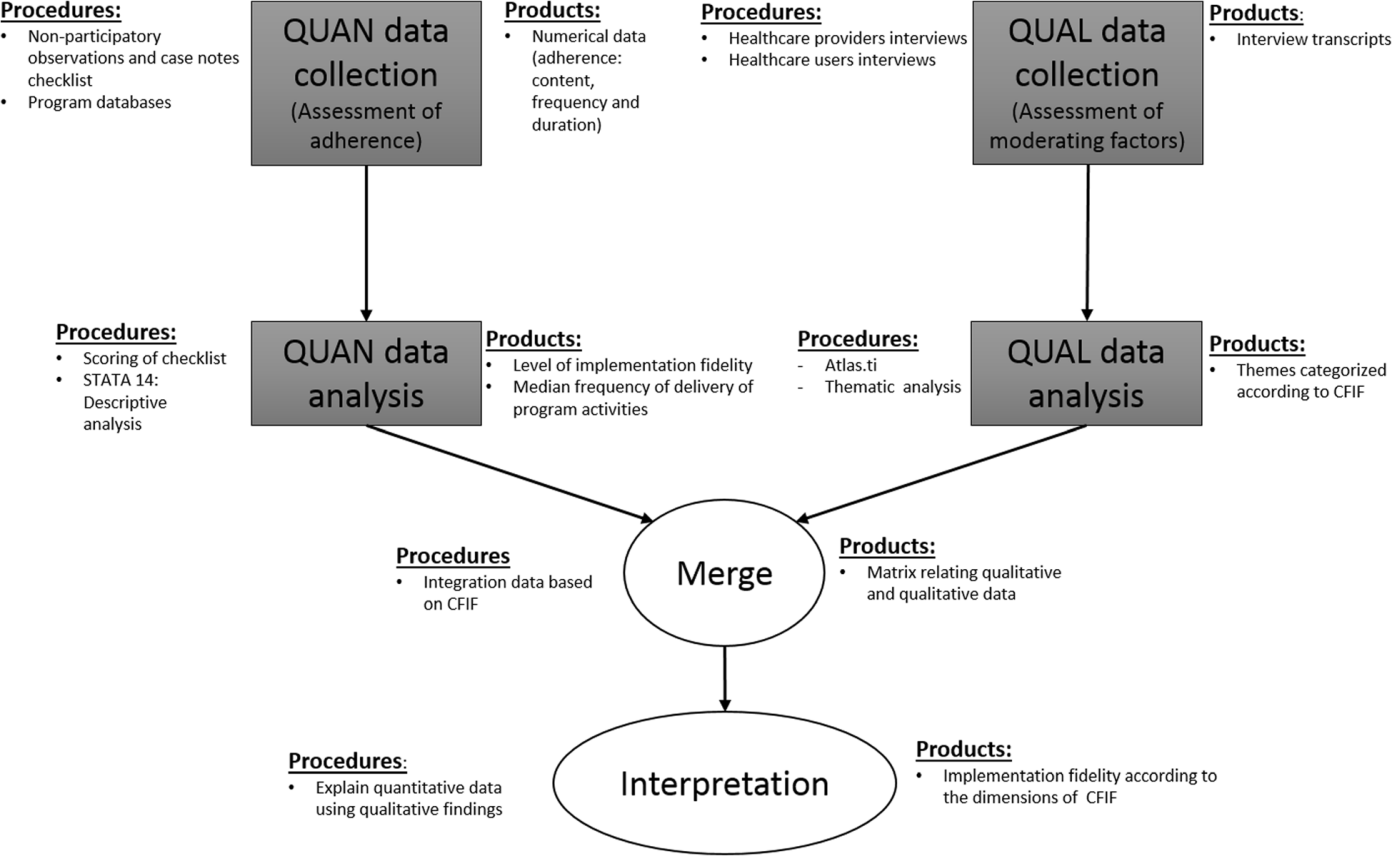
A convergent mixed method study to evaluate implementation fidelity of NUHS-RHS transitional care program

The study is a part of the realist evaluation of the NUHS-RHS [28]. In this study, we focused on the evaluation of the implementation fidelity of the NUHS-RHS post-discharge care program. A convergent parallel mixed methods study (Fig. 2) was used to guide data collection and analysis.

### Data collection and analysis

Data were collected between June 2016 to June 2017. Components of adherence including content, frequency, and duration were assessed using non-participatory observations, reviews of medical records and program databases. We were unable to examine coverage as there was no documentation of the total number of people who were offered the program. Moderating factors were examined using interviews with healthcare providers and healthcare users (patients and caregivers). Caregivers were interviewed as proxies to patients. As there was no benchmarking available within Singapore, we did not assess the quality of delivery and comprehensiveness of policy.

#### Development of a checklist

Program protocols were initially reviewed by study team members to provide an understanding of the program and how it aimed to achieve targeted outcomes. The intended components of the program identified were then validated against the program’s logic model, components of case management as proposed by the Kings’ Fund [29], and through conversations with a few care coordinators (CCs). After which, a list of intended components, including: (i) needs assessments, (ii) development of personalized care plans, (iii) care coordination (symptoms management, functional management, psychological support, medication management, home environment assessment, management of social issues, appointment management, promotion of self-care, referral to other services, advocacy and telephone monitoring), and (iv) case closure (complete discharge or discharge to less or more intensive care), was then developed as a checklist to assess the level of adherence to the program’s intention.

#### Assessment of adherence

Study team members conducted non-participatory observations of home visits and telephone monitoring – the core components of the program. Content of care delivery, interactions between healthcare users and providers, together with users’ responses to the service were observed and observation notes were taken. Medical records of patients enrolled into the programs were reviewed to provide a comprehensive picture of the services delivered throughout a patient’s healthcare journey from enrolment to discharge. Program databases comprised of records of patients enrolled in the program was reviewed to gather insights on the dose of the program.

Then, observation notes and reviews of medical records were assessed using the checklist by study team members. Program components were scored as “yes” or “no” for whether they were conducted or not. After which, level of adherence for each component of the program was calculated as the percentage of the number of cases in which specific component was scored with “yes” over the total number of observations conducted and medical records reviewed. Levels of adherence for specific component that fall within the 80–100% range were categorised as “high”, within 51–79% as ‘moderate’ and 0–50% as ‘low’ fidelity [30, 31]. In assessing dose of the program, frequency was computed for every patient as the summation of the number of times home visits and telephone monitoring were delivered throughout the program period. After which, median frequency for home visits and telephone monitoring was tabulated. Duration of individual patient enrolment was measured in days by subtracting the date of discharge from the program from the date of enrolment into the program. Then, the mean duration and standard deviation were tabulated accordingly.

#### Assessment of moderating factors

A convenience sampling of healthcare providers who were involved in the planning, development, and implementation of the program were recruited into the study. A contact list of all healthcare providers involved in the program was first obtained from the program manager. Based on which, invitation emails were sent out to recruit study participants. Only those who responded to the email invitations and agreed to be audio-recorded were interviewed by the study team member(s). In every interview session, an interview guide developed for the study (Additional file 1) was used to examine coverage of the program, content and moderating factors which may have contributed or hampered the implementation of the program.

Similarly, a convenience sampling of healthcare users including patients and their family members who have had experiences with the program for at least 3 months were recruited through their respective care coordinator (CC) to take part in the interviews. Eligible healthcare users who agreed to be audio recorded were eventually interviewed. In every interview session, content of program and their responses to program were assessed using an interview guide for healthcare users (Additional file 1). PS and MN conducted the interviews that lasted between 30 and 90 min. All interviews were audio-recorded, transcribed verbatim and coded thematically. Some healthcare users’ interviews were conducted in other non-English languages (Mandarin/Malay). They were transcribed in English, and analyzed thematically in two steps. The first step consisted of a deductive analysis, coding units of data according to the modified CFIF. Themes were categorized based on moderating factors defined. We further classified contextual factors under healthcare users, providers, and organizational factors. This was followed by an inductive analysis, seeking to elicit new themes or unexpected findings beyond the modified CFIF through coding and categorizing.

#### Data integration

Data from the various sources were given equal weightage, and merged by PM and MN at analysis stage using the modified version of the CFIF [32, 33]. An Information matrix was developed and used to summarize study findings. Qualitative themes were used to explain quantitative adherence data.

## Results

### Data sources

Forty-two (42) non-participatory observations were conducted, 29 at first-time home visits, 11 during follow-up visit and 2 during phone monitoring to assess adherence. For the same reason, medical records of 44 patients who were enrolled into the program were also reviewed. Out of the records reviewed, documentations regarding the program could not be found in 11 of them. Therefore, only 33 reviews of medical records were included in the assessment of adherence. Program databases containing a total of 2705 records were reviewed to determine the dose (frequency and duration) of the program.

For the assessment of moderating factors influencing program implementation, 25 healthcare providers including CCs, managers, and physicians involved in the implementation of the program and 45 healthcare users (patient/caregiver) enrolled in the transitional care program were interviewed.

### Adherence

#### Content

As illustrated in Table 1, most (10 out of 14) components of the program were found to be implemented with low and moderate level of fidelity.

**Table 1:** Implementation Fidelity (content) of NUHS-RHS transitional care program (based on 42 observations and 33 reviews of medical records)

Content	Adherence (%)	Level of Implementation Fidelity
Needs assessments	100.0	High
Development of personalized care plans	91.8	High
Symptoms management	89.2	High
Functional management	59.5	Moderate
Psychological support	43.2	Low
Medication management	63.5	Moderate
Home environment assessment	56.8	Moderate
Management of social issues	58.1	Moderate
Appointment management (e.g. Reminders, checking attendance and consolidation)	50.0	Low
Promotion of self-care through education and empowerment	87.8	High
Referral to other services	23.0	Low
Advocacy	16.2	Low
Telephone monitoring	77.3	Moderate
Case closure	50.0	Low

#### Dose (frequency and duration of enrolment)

The frequency of actualization of program specific activities was found to vary according to the needs of users, availability of CCs, and their confidence in delivering particular program components. For the period of program enrolment, a median frequency of 2 and 7 were found for home-visits and phone-monitoring respectively. Time spent on home visits was not explicitly tracked while a median of 10 min were found to be spent on every phone call monitoring. Patients were found to be enrolled in the NUHS-RHS post-discharge care program for a mean duration of 112.8 days with a standard deviation of 145.9 days.

### Moderating factors

**Fig. 3:**
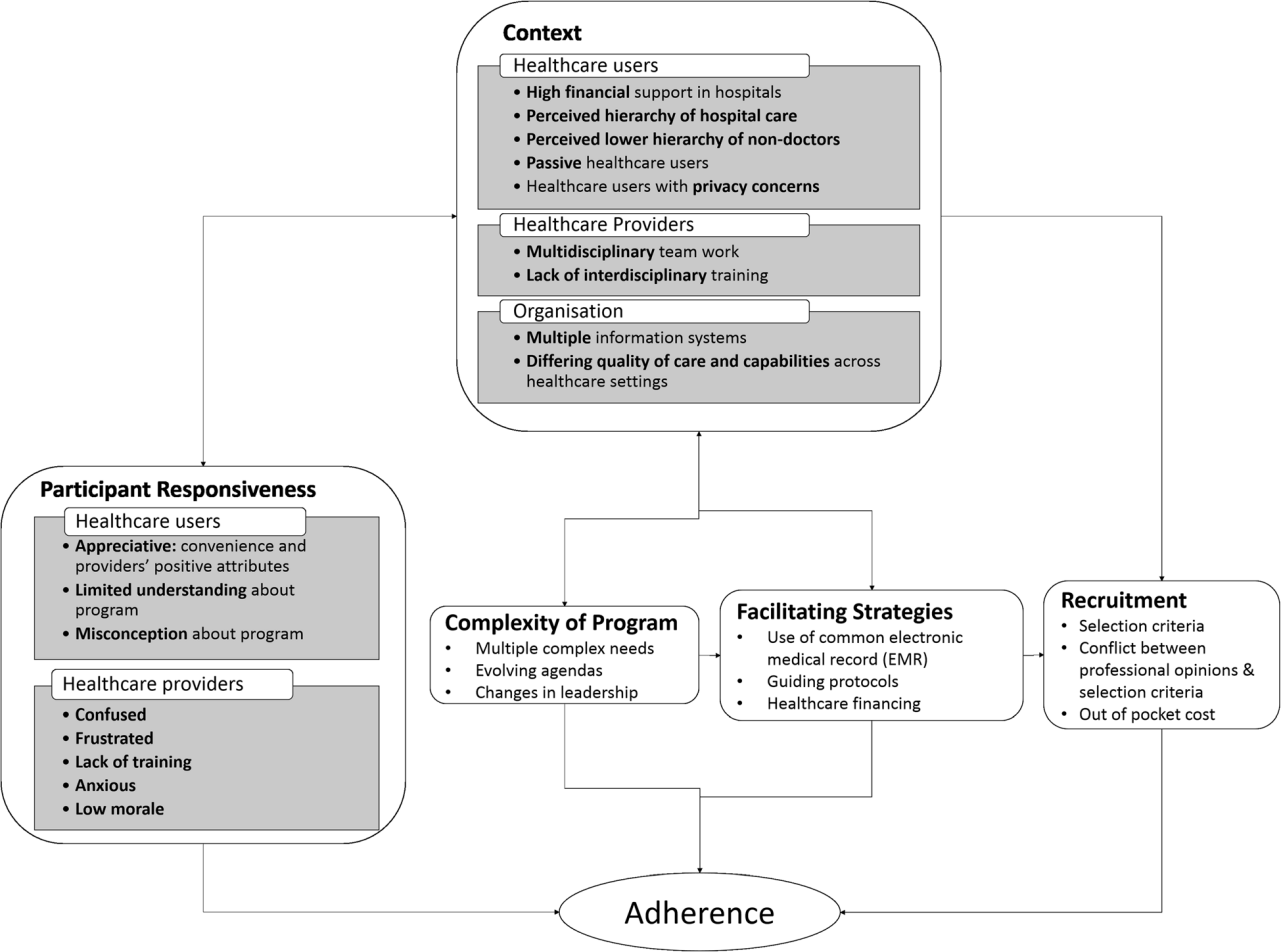
Moderating factors influencing implementation fidelity of NUHS-RHS transitional care program

Our study findings confirmed interrelated influence of moderating factors identified by the modified CFIF on implementation fidelity of the NUHS transitional care program (Fig. 3). A summary of key themes and their corresponding exemplary quotes is described on Table 2.

**Table 2:** Thematic findings and exemplary quotes

Moderating factors (level)	Themes	Exemplary quotes
Participant responsiveness: Users	Appreciative: convenience and providers’ positive attributes	*“(The CC is) very friendly, very approachable, and helpful. You can ask them questions and they have answers.” (Patient)*
	Limited understanding of the program	*“I think they (patients) are still not sure of this program yet. This program is about 2 to 3 years old. So it’s quite new I don’t think people take note that there is such home care service.” (CC)*
	Misconception of program	*“I don’t know whether how satisfied patient and family are. Sometimes, they really think that the home visit is just ‘trying to disrupt my usual routine’.” (CC)*
Participant responsiveness: Providers	Confused: regarding the program direction	*“We don’t have the clear guidelines. My departments doesn’t know what is coming is next. You do not want to walk a journey or road where you don’t know where it is leading to.” (CC)*
	Frustrated: impossible to avoid hospitalization	*“Elderly patients are more fragile, they get broken skin and more delicate. They are also more susceptible to side effects of the chemotherapy and the drug that we are giving them. So then in this case is we educate family on how to take care of the symptoms. When patient has fever, (drowsiness) … we (call) them few times to bring him in. This is an example we cannot avoid hospitalization.” (CC)*
	Lack of training: communication and psychological issues	*“We are not trained to (do) psychological assessment. Actually our boss thinks that we should be trained so he has actually liaised with the psychological department but they are still liaising.”(CC)*
	Anxious: Job security	*“The security of our job is very real (concerns) to us … If the program suddenly shuts down, where can we go?” (CC)*
	Low morale: no confidence or satisfaction	*“You don’t feel the sense of confidence, you don’t feel satisfied because you feel that this piece of work you are doing can close up anytime you know. In the end they (client) die natural death.” (CC)*
Complexity of program	Multiple complex needs: require many components of interventions	*“The patients need intensive rehabilitation because: i. their medical conditions are very complex, ii. they may or may not have severe illnesses, iii. They have combination of many chronic diseases so families can’t manage.” (Program manager)*
	Evolving agendas: changes in recruitment criteria	*“They actually changed their recruitment criteria. Initially, they mentioned that as long as a patient has 3 admissions in any hospital, then we can recruit. But now they change it – out of the 3 admissions, at least have one admission must be in National University Hospital – then we can recruit the patient.” (CC)*
	Changes in leadership: changed program direction	*“We have done this model and got used to it. So suddenly there’s (new leadership) and a new model coming. Any changes (in the model) people are like, “oh what is our position?” We are still unclear, where should we place ourselves, so that makes people think" where’s the future?” (CC)*
Facilitating strategies	Use of common electronic medical records (EMR)	*“We (the healthcare providers) share the same electronic platform … physicians can look through the system at all the investigations (done for the patients).” (Physician)*
	Guiding protocols	*“We have a checklist to go through the process, standardized protocol. So we started with in-patient, (do) the assessment, then each component (like) medical, social.” (Program manager)*
	Healthcare financing: funding and subsidy	*“There are two main sources of funding, there is program funding and “get well” funding. Based on the means testing levels, there is the corresponding funding or (subsidy).” (Physician)* “*This program is heavily subsidised, so typically every week, the full cost will range to be about $600 weekly, so after subsidy it comes out to $80–100. For this program, majority of the patients, we are charging them about 20% of the cost, and the remaining is actually funded through our Ministry of Health funding sources … For needy patients, they would not need to pay anything.” (Program manager)*
Recruitment	Selection criteria: to guide recruitment	*“If the patient has 3 times or more admissions, then we will enroll the patient. Of course we will have to ask (for) patient’s permission.” (CC)*
	Conflict in professional opinions of CC and the program selection criteria	*“Ok, this one (client has) limited mobility and I will say if patient gets home care will be better. It’s not like they cannot walk. (This homecare service is only for non-ambulatory) patients. But if you can pay a visit to them, they can do better during the time period and minimise the readmission.” (Allied health)*
	Out of pocket cost	*“The (out of pocket) cost of the services is the main barrier for the patients to enroll in the program, it needs to be revised to cater wider range of the patients.” (Program manager)*
Context: Users	High financial support within hospitals	*“They rather be admitted to the hospital, because in the hospital they are supported by MediSave (government medical saving scheme) and receive subsidised care.” (CC)*
	Perceived hierarchy of hospital care	*“I rather come here (to the hospital). The apparatus are all here, if any problem the doctors are around, so I think it’s easier to just come and sit and then do what you need to do.” (Patient)*
	Perceived lower hierarchy of non-doctors	*“Only the doctor will understand the situation and issues. I don’t think (the CC) will be able to describe the situation to me unlike the doctor who is helping us.” (Patient)*
	Passive healthcare users	*“So some patients are quite passive, are not concerned or update us on their conditions. When we call them and dig up the problems they have, it’s too late. You know every time we call, either the patients already have problem or the patients is already admitted.” (CC)*
	Healthcare users with privacy concerns - less likely to enroll	*“Because some clients are more private, they don’t like people to invade into their privacy, so they don’t welcome people to (their) house.” (CC)*
Context: Providers	Multidisciplinary team work: organizational and national focus	*“I think what has worked for us is that we have the support of all the other professions, the multi-disciplinary professions. Doctors at the clinics, the nurses at the clinics, the day rehab centre, the social workers coming into the picture. The doctors giving us input into how to manage better. And that network of services that we have.” (Social worker)*
	Lack of interdisciplinary training	*“We mainly focus on the medical aspects. So, if there are any social issues, and if we cannot handle, then we will tap on the medical social worker. We will bring in our colleagues.” (CC)*
Context: Organization	Multiple information system	*“Currently we are (using) CDOC for documentation and our clients are enrolled in CCMS (another system) which is from MOH. We have to enter patient into CCMS that means it is enrollment to the program, but the thing is CCMS is not linked to what we are using currently so it is actually very challenging for us.” (CC)*
	Differing capabilities across healthcare settings	*“I think we have some service gap in the community (providers). Some of them (do not cater) especially for dementia patient (as) they have behavior issue.” (CC)*

CC: Care coordinator

#### Participant responsiveness

The majority of healthcare users were observed to be responsive and had good rapport with CCs during home visits. They expressed *appreciation* of the program and viewed the *convenience* of having *empathetic, caring, proactive and knowledgeable CC* visiting them at home to be most valuable. Nonetheless, the extent of responsiveness was found to fluctuate according to level of awareness. Some healthcare users were found to have *limited awareness* of the specific services the program offered, its goals and the role of the CC. Therefore, there was *misconception;* users regarded home visits as “disruptions” with *little value* and CCs as “doctors’ assistants” with limited capability, leading to lowered perceived value of the program. In contrast, those who valued team-based and patient-centric care were more responsive.

Healthcare providers reported initial excitement about the prospects of the program and believed that the program would enhance transition and recovery of patients. However, the level of healthcare providers’ responsiveness changed over time due to *confusion* created by lack of well-developed program direction and protocols. Expectation to cope with increasing workload, yield positive outcomes within a short time and lack of training, created *anxiety* among healthcare providers. Uncertainty surrounding the future direction of the program and job security of the CCs perpetuated *frustrations* among them. Altogether, these factors *lowered morale* and lowered providers’ responsiveness.

#### Complexity of program

The *myriad complex needs* driving the development of the program, heterogeneity within the healthcare users that the program serves, detail of program components, and involvement of numerous actors with different perspectives rendered the program complicated. While it would have been ideal to have a simple, standardized, and specific program protocol, it was impossible to do so given many moving parts. *Changes in leadership* were found to shape the behaviour of different actors of the program, requiring successive adaptations throughout the implementation. For example, throughout the short implementation period, revisions to enrolment criteria, recruitment strategies, and roles of different providers were made due to the change in leadership.

#### Facilitating strategies

The use of *a common information system*, *guiding protocols* and *financial support* were found to facilitate the implementation of the transitional care program.

All healthcare providers involved were granted access to the common electronic medical records (EMR) which functions to systematically consolidate medical information and allows sharing of information between actors across disciplines and care settings. Although the shared EMR has successfully facilitated information continuity to a great extent, its wider adoption remained a work in progress. Having to manage other work pressures, documentation was perceived by healthcare providers as *time-consuming additional administrative* work, and hence not prioritized. Besides the common EMR, a standardized care needs assessment and referral system was implemented to help facilitate appropriate placement of patients for intermediate and long-term care (ILTC) in the community. However, healthcare providers experienced difficulties in using this system, forcing them to manually search for and refer patients to ILTC after the program.

Guiding protocols describing selection criteria and key steps to be taken in delivering program were developed at the beginning of the program. Protocols guided healthcare providers to standardize the delivery of program and manage patients at home independently or as a team. However, there were different levels of granularity in the details available for various program components within the protocols. Detailed description was available only for needs assessments, development of care plans, and promotion of self-care. Consequently, healthcare providers had to improvise for other components and dose of the program based on their experiences. This caused discomfort among healthcare providers as many of them were used to working within the hospitals where standard operating procedures (SOPs) were clearly defined. The lack of specific details related to referrals to other services had also resulted in difficulties in referring users to other community services.

Our study revealed that block funding provided by the Ministry of Health (MOH) Singapore which covered healthcare manpower costs, transport fees, training expenses, and service costs of providing the program facilitated the implementation of the program. Subsidies available for running the program lowered the barrier for adoption as users had to pay minimal fees for the program.

#### Recruitment

Wrong and late referrals were noted to adversely affect recruitment in the early phase of the program. This was quickly resolved through continuous engagement of healthcare providers who refer patients by the program team and clarification of selection criteria. Later, healthcare providers experienced *difficulty in reconciling the differences between their professional opinions and the program selection criteria* in recruitment of patients. In response to this, exceptions with justification often had to be made.

Approximately 10–20% of the eligible patients refused to participate in the program. Those who did not appreciate the value of the program and had *privacy concerns*, were reported to more likely to decline participation. *Out of pocket costs* were also found to be the other reason for patient refusal.

#### Context

At users’ level, having been used to a largely hospital-centric system with easy access to specialist care, the healthcare users preferred to be cared for in the hospitals. Hospital care was highly subsidized, making it more appealing to users compared to community care. Non-doctors and professionals providing care outside of hospitals *were also often perceived as inferior to doctors* working in the hospitals. This was perpetuated with *passive culture* among users which contributed to the belief that it was providers’ responsibility to assist in the management of their health.

At the providers level, good progress in *multidisciplinary collaboration* in the delivery of the program was noted where physicians, nurses, and allied health professionals worked well together. This, in turn, contributed positively to the implementation of the program. Conversely, even with the focus to provide holistic care for individuals with complex needs, the phenomena of “super specialization” in which healthcare providers specialize in an organ or certain aspect of patient care persisted. There was limited *interdisciplinary training* among providers. This was problematic as diversification of professions and their specialized tasks contributed to slow progress towards provision of holistic care. Also, this affected providers’ ability to adequately manage individuals with multiple needs, causing delays in care. In many occasions, CCs had to seek help from social workers from the hospitals as they were not trained and therefore not confident in managing social issues and advocating for users.

At the organization level, fragmentation was found in information systems, with varied level of adoption and sub-systems adopted by different healthcare providers. *Requirements to document on multiple systems* was a great concern to healthcare providers as it consumed more time. Fragmentation in *care capabilities* among providers were also observed. This was largely contributed by resource constraints at the community level. In addition, the formal organization of and practices within community care in Singapore were less developed compared to the hospitals, affecting their capability to collaborate. In many instances, CCs could not discharge patients to the community providers within the NUHS-RHS after the planned program period as the community care providers did not have the capability to continue management of care.

## Discussion

We examined the implementation fidelity of a transitional care program to integrate services for high need individuals in Singapore after discharge from hospital, using the modified version of CFIF.

Our findings revealed several contributing factors to variability in implementation fidelity of components of the NUHS-RHS transitional care program. These included: (1) complexity of the program, (2) extent of facilitation through guiding protocols, (3) facilitation of program implementation through CCs’ level of training and confidence, (4) evolving healthcare participant responsiveness, and (5) the context of suboptimal capability among community providers. Our results showed these factors influenced implementation fidelity of the program in an interrelated manner.

We found that healthcare providers experienced the program as a complex intervention predominantly due to difficulties faced in its implementation and its intention to be “patient-centric”- i.e. designed and organized around the needs of the healthcare users (patients and caregivers). Findings highlighted heterogeneity within the users in terms of needs and responsiveness, making it impossible to standardize program implementation. Changes that occurred over time always required personalization of care. Personalization actualized in the form of continuous adaptations in response to changing needs through revising goals and working towards what is best for and preferred by users. This was reflected as variation in implementation fidelity of the program. Similar findings were reported by Muntinga et al. [34], who assessed implementation fidelity of a complex intervention of a chronic care model for frail, older people living at home. They highlighted the importance of careful interpretation and consideration of misinterpreting improvement to a program with low/non adherence. For the same rationale, even when fidelity and adaptation were historically considered as contradictory, reconciliation between these two aspects has increasingly been advocated [35].

The high level of fidelity found for needs assessment, development of care plans, symptoms management and promotion of self-care was due to the availability of detailed description of protocols for these program components. Providing care outside of the hospitals was new for most CCs involved in the program, thus, detailed protocols gave guidance for CC to confidently deliver care on their own and to standardize delivery of care. This finding is in line with prior studies which suggested that detailed protocols facilitated implementation of program. Another explanation for high implementation fidelity is CCs’ training and confidence. CCs were trained in the hospitals and therefore familiar with medically-oriented tasks such as needs assessment, development of care plans, symptoms management, and promotion of self-care. It was not surprising that CCs naturally prioritized tasks which they were familiar with.

Low implementation fidelity found for psychological support can be explained primarily by the lack of interdisciplinary training and experience among providers involved in the program in providing this support. CCs were found to be still accustomed to providing medical care in the hospital environment and lacked training in management of psychological issues. As highlighted by Ong et al. [36], this is likely contributed by how medical education is delivered in Singapore. Currently, medical education stresses on specialization of physical health with little focus on psychological health, discouraging cross training of providers and impairing the capability of healthcare providers to provide holistic care [36]. Without healthcare providers who are trained to provide holistic healthcare, the transitional care program cannot be optimally delivered. Users’ low responsiveness observed in terms of the lack of awareness, misconception about the program and perceived hierarchy of profession might have also hindered the disclosure of psychological issues. Psychological support was probably thought to be beyond the scope of the program and capability of the CCs. Moreover, given that users displayed little appreciation for patient-centric care, it was also likely that they would prefer to see mental health specialist for their psychological issues. This is consistent to previous conclusion by Storm et al. [37]. Healthcare users’ characteristics and level of engagement were identified to be pivotal in implementation of transitional care program.

The Singapore mental health study revealed a huge treatment gap for mental illnesses [38]. Results from their follow-up studies consistently suggested that low mental health literacy and high stigma against mental illness found to be more pertinent among the older population to be the main barriers to seeking treatment [39–41]. Therefore, it was likely that older adults who were enrolled in the program avoided disclosing their psychological needs and seeking treatment due to low mental health literacy and high stigma. This highlighted a gap in management and an urgency to provide holistic care considering all aspect of users’ needs as there is growing prevalence of concurrence of physical and psychological issues which requires holistic care [42].

Our results showed a lack of referrals to other services by CCs was due to: i) the lack of specific details guiding CCs on where to refer patients to, and ii) difficulties faced by CCs in using the common referral system. This was worsened by providers’ diminishing responsiveness to the program. As providers became less responsive, they were less likely to take a proactive role in overcoming the issues faced. Furthermore, suboptimal capabilities found among community providers were found to deter referrals to other services. For this reason, CCs were often forced to continue to manage patients after the planned program duration. A study by Hasson et al. suggested that providers’ responsiveness is a key driving force for achieving high implementation fidelity. In their study, frustrations associated with limited facilitation in the study was found to ease quickly with high providers’ responsiveness [21].

We found the lack of interdisciplinary training and diminishing responsiveness among CCs to be the main reason behind low implementation fidelity of advocacy and appointment management. With the lack of interdisciplinary training, CCs were not comfortable in delivering these services as they were traditionally specialized by other providers. Advocacy was typically delivered by social workers while appointments were managed by other designated operation teams in the hospitals. Consequently, CCs often had to rely on the assumptions that advocacy and appointment management would have been delivered by other providers (within the hospital), since patients enrolled in the program continued to receive care from the hospitals.

### Key implications and recommendations

Based on our study findings, we propose several generalizable recommendations for decision makers to consider when designing and implementing a transitional care program as part of a strategy for integration of healthcare services. Firstly, ex-ante, efforts should be put in place to improve the responsiveness of both healthcare users and providers about the program. There should be better alignment in expectations between both parties. Secondly, the capability building of community care providers should be prioritized.

For better alignment of expectations between users and providers, efforts can be put into increasing users’ awareness of the program and addressing misconceptions, roles of CC and community care. Thorough explanation by the CCs about the program and their roles together with intentional involvement of users in making shared decision related to their care can be the potential solutions. However, as the willingness, readiness, and ability of healthcare users to participate were demonstrated to be the pre-requisites required for engaging patients who were receiving transitional care programs [43], future programs may wish to assess these factors to determine who should be enrolled into the program. This would more likely lead to high users’ responsiveness.

In addressing the diminishing responsiveness of providers, appropriate incentives (financial and others) can be put in place to motivate healthcare providers. Emphasis should also be made for providers to have more active involvement in the design and development of programs. Such a strategy was found to secure the commitment of providers, improve motivation, and ensure sustainability in implementation of a similar program [44]. It can also be expected that bigger involvement will add confidence among providers and facilitate clarification of doubts related to the implementation of the program. Also, to augment the success of the program, engagement of healthcare providers should also be approached from the policy perspective. In doing so, Sturmberg and Lanham proposed for a complex adaptive policy framework with loose boundaries that facilitate adaptability and allow emergence of optimal solutions best fitted for each unique care landscape [45]. Improvements focused on interdisciplinary training should also be prioritized. This could be approached through didactic and experiential interdisciplinary healthcare education which has been shown to be beneficial for equipping healthcare providers with relevant skills to improve patient outcomes, especially for older adults with complex needs [46, 47].

To further develop capability among community providers, the hospitals that have more resources can possibly take a more pro-active role in training the community care providers. Community care providers could also be empowered by making changes to the current healthcare financing model to support integrated care interventions like transitional care programs. Rather than charging patients based on episodes of care and provider type, a financing mechanism that follows the patient and incentivizes quality and continuity of care should be put in place to pool charges across services and providers.

### Strengths and limitations

To the best of our knowledge, this is the first study that has taken the opportunity to assess context-specific implementation process of a transitional care program in the context of Southeast Asia. Particularly in the context of Singapore, where most of previously conducted evaluation on similar programs evaluation [18, 24–26] focused mainly on assessing the effectiveness, our study provided important insights to facilitate further development and scaling up of such a transitional care program within NUHS-RHS and beyond.

We used a validated framework, the modified CFIF, which has been used to assess implementation fidelity of complex healthcare programs [21, 22]. Considering the intricacy of the program with multiple components, the modified CFIF provided a useful framework to direct the assessment of specific factors that have been found to influence implementation fidelity of complex programs. This allowed the identification of specific areas for improvements and comparison with other studies that had used the same framework. However, we were faced with challenges in the measurement of the dimensions of adherence, quality of care, and comprehensiveness of policy due to the lack of program level data and variability in the delivery of the program. As providers identified documentation to be resource-intensive and time-consuming, improvement in data collection methodologies should be explored to ensure that comprehensive and accurate data are captured in the future.

Measurement of adherence was challenging as the basis of assessment could not be standardised and explicitly defined due to the nature of the program. This is consistent with other studies that have attempted to examine the implementation fidelity without clearly defined reference points of measurements [21, 34]. Keeping reference points for the measurement of adherence broad and revisiting them with the evolution of the program could be explored to solve this issue. To achieve this, it would be essential for evaluators to work closely with program implementers so as to ensure that evaluators are kept updated of changes of the program.

Comprehensive and accurate data gathered using multiple methods during implementation of the program added increased credibility to the study findings as they reflect the actual circumstances in which the program was being implemented. Nevertheless, it must be acknowledged that because this study is a cross-sectional study, it did not take into account the changes of the program over time. We learned that changes occurred but details of the changes remained unknown. Therefore, we were unable to explicitly point out the effects of the evolution on implementation fidelity of the program. For a more comprehensive assessment of implementation fidelity, longitudinal investigations should be conducted in the future to understand how implementation of the program changes over time.

In addition, only users who were part of the program were interviewed as it was operationally challenging to recruit those who rejected enrolment into the program. For simplicity, we combined findings from patients and their caregivers. The literature highlighted distinction between the roles of patients and caregivers in transitional care [37], therefore, it may be valuable to evaluate patients’ and caregivers’ views separately. Such analyses could also be useful in tailoring specific interventions for patients and caregivers.

## Conclusion

In assessing the implementation fidelity of the NUHS-RHS transitional program, we found detailed protocols coupled with CCs’ level of confidence and familiarity in specific program components to contribute to high implementation fidelity. In contrast, program complexity, diminishing participant responsiveness, lack of specific training for CCs, and limited capability of community providers have observably made the implementation of the program challenging. Such findings highlighted the need for greater focus on engaging both healthcare providers and users, training CCs to equip them with relevant skills required for their jobs, and further development of the capability of the community providers. Finally, as illustrated by the Rainbow Model of Integrated Care [48], our study re-iterated the importance of fostering integration at the micro- clinical level, the meso- professional or organizational level and the macro or systemic level in order to achieve desired outcomes.

Other health and social care networks within Singapore and abroad, alike to the NUHS-RHS, are experimenting with new integrated care models including transitional care programs. Findings from existing research of the (cost-)effectiveness of integrated care are to a major extent inconsistent because of the variations in the strategic outcomes, methods of implementation, contexts (i.e. system and policy) and/or applied evaluation measures [49]. The findings from this study fill some gaps in current research, by evaluating the implementation fidelity of the NUHS-RHS transitional care program using a rigorous and comprehensive design that balances the needs of context-specific evaluation. This study may also provide relevant insights to those involved in the development, implementation and/or evaluation of transitional care programs as part of integrated care initiatives.

## Additional file

Additional file 1: Topic Guide for Interviews. (DOCX 18 kb)

## Abbreviations

CC: Care Coordinators
CFIF: Conceptual Framework for Implementation Fidelity
EMR: Electronic Medical Records
FAs: Frequent Admitters
ILTC: Intermediate and Long-term Care
MOH: Ministry of Health (Singapore)
NUHS: National University Health System
RHS: Regional Health System
SOPs: Standard Operating Procedures
