# Supplementary material for: Implementation fidelity of a strategy to integrate service delivery: learnings from a transitional care program for individuals with complex needs in Singapore
Source: BMC Health Serv Res. 2019 Mar 19;19:177. doi: 10.1186/s12913-019-3980-x (PMC6425607; doi:10.1186/s12913-019-3980-x)
Supplement: Supplementary file 1 — Topic Guide for Interviews. (DOCX 18 kb) [file 12913_2019_3980_MOESM1_ESM.docx]

**Annex 1: Topic Guide for Interviews**

**A. FOR HEALTHCARE PROVIDERS**

1. Adherence
   1. **[Coverage & Recruitment]**

- How were patients enrolled into the programs?
- What proportion of eligible patients were enrolled into the program?
- Did you face any difficulty during recruitment? What were the challenges you faced during recruitment?

**1.2 [Content]**

- To what extent were the activities implemented as planned?
  - What were the activities included as part of the program?
    - Who were involved in the activities?
    - Besides what were included in the program, did you arrange other services for patients? If yes, please tell me more about them.
    - Did you face any difficulty in the delivery of the program? If yes, please share the challenges you faced.
  - Did you involve the patients and (or) their caregivers during the programs?
    - How did they respond to the program?

1. **[Moderating Factors]**
   - What were the key success factors of the implementations of the NUHS Transitional care programs?
   - What were the challenges faced during the implementation of the programs?
   - Did you feel prepared to do what was expected of the program?
   - Were the cultural, social, organizational and political factors supportive of the implementation of Transitional care program? What makes you say that?
2. Any other comments about the transitional care program?

**B. FOR HEALTHCARE USERS (PATIENTS OR PROXIES)**

**[Content]**

1. When you or [patient’s name] were discharged from the hospital, how was your transition prepared? How were you followed up?
2. Is there a person/people who help you or [patient’s name] organize your healthcare as part of the program?
   - - - If yes, what kinds of help have you and your family members received from your care coordinators [CC’s name]?
       - How that did affected your care?
3. How have you and family members been involved in your treatment?
4. In general, do your healthcare providers including doctors, nurses, therapists, social workers and care coordinators work together? Please tell me how they work together

**[Participant Responsiveness]**

1. On a scale from 1-10, how satisfied are you or [patient’s name] with the care received?
   - What makes you give this rating?
   - Would you or [patient’s name] recommend the care received to your friends or family or use the same care as received if you are offered it in the future?
2. What do you or [patient’s name] like best about the service provided by the [name of CC or program]?
3. What do you or [patient’s name] not like about the service provided by the [name of CC or program]?
4. How can the program be improved?
5. Any other comment about the transitional care program?
